# ‘It is human work’: qualitatively exploring community roles that facilitate cultural food security for people from refugee backgrounds

**DOI:** 10.1017/S1368980024000326

**Published:** 2024-02-06

**Authors:** Tina Gingell, Rishita Adhikari, Nehal Eltahir, Fulgence Ntahomvukiye, Evelyn Pe, Kate Murray, Ignacio Correa-Velez, Danielle Gallegos

**Affiliations:** 1 School of Nutrition and Exercise Sciences, Queensland University of Technology (QUT), Brisbane, Australia; 2 Centre for Childhood Nutrition Research (CCNR), Level 6, 62 Graham Street, South Brisbane, Qld 4101, Australia; 3 Islamic Women’s Association of Australia, Brisbane, Australia; 4 Ethnic Communities Council of Queensland, Brisbane, Australia; 5 School of Psychology & Counselling, Queensland University of Technology (QUT), Brisbane, Australia; 6 School of Public Health & Social Work, Queensland University of Technology (QUT), Brisbane, Australia

**Keywords:** Participatory action research, Co-design, Strength-based, Refugee, Community research, Cultural food security, Food sovereignty

## Abstract

**Objectives:**

Cultural food security is crucial for cultural health and, for people from refugee backgrounds, supports the settlement journey. Cultural communities are vital in facilitating access to cultural foods; however, it is not understood how refugee-background communities sustain cultural food security in the Australian context. This study aimed to explore key roles in refugee-background communities to understand why they were important and how they facilitate cultural food security.

**Design:**

Interviews were conducted by community researchers, and data analysis was undertaken using best-practice framework for collaborative data analysis.

**Setting:**

Greater Brisbane, Australia.

**Participants:**

Six interviews were conducted between August and December 2022 with people from a refugee-background community, lived in Greater Brisbane and who fulfilled a key food role in the community that facilitated access to cultural foods.

**Results:**

Fostering improved cultural food security supported settlement by creating connections across geographical locations and cultures and generated a sense of belonging that supported the settlement journey. Communities utilised communication methods that prioritised the knowledge, wisdom and experience of community members. It also provided community members with influence over their foodways. Community leaders had an ethos that reflected collectivist values, where community needs were important for their own health and well-being.

**Conclusions:**

Communities are inherently structured and communicate in a way that allows collective agency over foodways. This agency promotes cultural food security and is suggestive of increased food sovereignty. Researchers and public health workers should work with communities and recognise community strengths. Food security interventions should target cultural food security and autonomy.

Foods are essential for life, as they provide nourishment for the mind, body and spirit. Foods sustain us physically through the micro and macro nutrients contained in the food. Foods, however, also have meaning beyond their biological components. Specific foods can link across time and space and as such cultural foods are interlinked with mental health, culture, identity and placemaking^(1–3)^. They bring about fond memories, create happiness and generate a sense of belonging^(1–3)^. For people from refugee backgrounds, the cultural and spiritual connection provided by food can support and enhance successful settlement in a new environment^(1,3,4)^.

Food security ‘exists when all people, at all times, have physical, social and economic access to sufficient, safe and nutritious food which meets their dietary needs and food preferences for an active and healthy life^(5)^.’ The inclusion of ‘food preferences’ in the definition indicates that cultural foods are an aspect of food security. In other words, having access to a sufficient quantity of foods does not necessarily mean people are nourished for an active and healthy life (nutritionally, spiritually and culturally). Power^(6)^ argued that the concept of food security was constructed without consideration of Indigenous perspectives, and that cultural food security (which includes access to foods harvested using cultural methods) were vital for some cultures. She stated that ‘food obtained from (cultural) food systems links the environment and human health, and forms the basis of social activity, social cohesion, and social integration^(6)^.’ This suggests that cultural food security is an important public health concept for community health and well-being, and consequently cultural food insecurity should be addressed by policy makers.

Since then, research has revealed that, especially for forcibly displaced communities, cultural food security is crucial for cultural health and survival and to facilitate the settlement journey^(1,2,6–9)^. In this sense, the settlement journey is about placemaking and creating a sense of belonging^(10)^. Two noteworthy examples highlight that culturally meaningful foods (hereafter cultural foods) and foodways enable settlement for refugee-background communities. Foodways encompass the growing, purchasing, preparing and consuming of foods within the community, and therefore incorporate cultural, social and economic food practices^(11)^. In Brazil, two internally displaced communities (Pataxó and Pankararu peoples) banded together to purchase land for cultivating^(7)^. These communities rebuilt food traditions (cultivation, cuisine and rituals), restored culture and resisted the economic and political forces that caused their displacement. A new village (Cinta Vermelha-Jund) was established, and community members were able to permanently settle. In Coffs Harbour, Australia, a community from Myanmar transformed front and backyards into gardens and established cultural food enterprises, thereby undergoing ‘place-making’ and gaining a sense of belonging^(1)^. When reporting on this, Hughes^(1)^ stated ‘[f]ood not only constitutes a living culture, through the act of recreating and sharing (cultural) foods, it also represents a way to engage with the present in a new environment^(1)^.’ These cases highlight that when displaced communities are able to act autonomously, new foodways may be formed to sustain cultural food security, and as a result, communities have the opportunity to flourish.

Communities acting together to form new foodways is indicative of collectivist values. These values are reflected in interdependence, and the goals of the group/community are prioritised over those of the individual^(12)^. Many people from refugee backgrounds, although not all, may come from cultures that retain these collectivist values^(12)^. Further, research has shown that displacement significantly disrupts social connections. Upon arrival in a new environment, people seek to form new relationships to create a sense of belonging^(13,14)^, and food is often used as a pathway to achieve these connections^(1,13)^. This indicates that even those not from collectivist cultures may be inclined to connect with and support others going through similar experiences, thus displaying collectivist attitudes. However, this may be at odds with the broader community when the dominant culture is individualistic, such as in Australia. These cultures value self-sufficiency and autonomy, and land and foods are often viewed as commodities^(15,16)^. It could be argued, therefore, that the collectivist action of refugee-background communities in creating new foodways sustains cultural food security in individualist societies.

There is a lack of understanding, however, about how refugee-background communities are inherently structured to sustain cultural food security in the Australian context. This paper reports on how communities build environments that facilitate cultural food security which supports settlement. In particular, it explores the roles of key actors and how they support cultural food security in refugee-background communities. Key actors include community members that fulfil an active food role in the community (e.g. farmers, grocery store owners, market stall owners, restaurant owners and community leaders). Consequently, the objectives of this study were to explore those key roles to understand why they were important.

## Methodology and methods

The project was approved by the QUT Human Research Ethics Committee (Project ID 5260).

### Methodology

The overarching research project, *Connecting with Cultural Foods*, aimed to improve food security for people from refugee backgrounds living in Greater Brisbane. The project was underpinned by participatory action research methodology^(17)^. This methodology draws on lived experiences to generate new knowledge about how political and social structures affect experiences^(17–19)^. It aligns with collectivist ideals and suits cross-cultural research by incorporating community member voices into the research agenda, by involving them in all aspects of the research^(18,20)^. The *Connecting with Cultural Foods* research team included four academic researchers (TG, KM, IC-V, DG) and five community researchers. Community researchers were employed community members that had a lived refugee experience or worked closely with people that did and represented a range of cultures that live in Greater Brisbane (Sudanese, Afghan, Burundian, Burmese and Nepali). Community researchers were involved in designing the data collection tools, conducting interviews, analysing and interpreting the data and disseminating the findings, and four were also involved in reviewing the manuscript and are named as authors in this paper (RA, NE, FN and EP).

### Methods

#### Participants and recruitment

Two previous project activities (co-design workshops and survey) informed the recruitment of interviewees. Between January and April 2022, the overarching project held co-design workshops, with eight refugee-background community members. Attendees mapped their personal journeys accessing cultural foods in Greater Brisbane, Australia, and collaboratively identified five community roles which were crucial to facilitating access to cultural foods. These roles were: community leaders, grocery store owners, restaurant owners, market stall owners and farmers. Between May and July 2022, the overarching project then conducted a survey of community members (*n* 157). The survey asked participants to nominate businesses and locations where they physically sourced their cultural foods, such as grocery stores, restaurants, butchers and farmers markets. Community members were also asked to nominate other community members that facilitated access to cultural foods, such as providing information on where to source foods or knowledge on how to prepare cultural foods. Survey data were collated and sorted into the five community roles identified during the co-design workshops. The list of names/businesses from the survey data was then sorted by culture and frequency of nomination by community members in the survey. The lists were examined against the inclusion criteria, and one business/person was selected from each community role, ensuring that a range of cultures and geographical locations were represented. The data from the co-design workshops and survey were used for recruitment purposes and were not included in the analysis.

This paper reports on interviews undertaken subsequently with community members fulfilling the five community roles identified in the co-design workshops. The inclusion criteria were people who (1) came from a refugee-background community and lived in Greater Brisbane and (2) fulfilled a key food role in the community that facilitated access to cultural food. A refugee-background community was any cultural community that included members with a lived refugee experience, but it was not a requirement that key actors themselves had a lived refugee experience. According to the 1951 United Nations Refugee Convention, a refugee is ‘someone who is unable or unwilling to return to their country of origin owing to a well-founded fear of being persecuted for reasons of race, religion, nationality, membership of a particular social group or political opinion^(21)^.’

Businesses (grocery store owner, restaurant owner and market stall owner) were contacted by the first author and/or a community researcher attending the workplace and asking to speak to the owner. Where the owner was contactable, they were provided with participant information, invited to participate and a face-to-face meeting was organised. People (community leader or farmer) were contacted by phone and a face-to-face meeting was organised where information about the project was provided. When a person was uncontactable or declined to participate, another business/person was selected from the list and the same process followed, until one person was recruited for each community role.

Six interviews were conducted between August and December 2022. Four interviewees (67 %) had a lived refugee experience, one had an intergenerational refugee experience (i.e. their parents had a lived refugee experience), and it was not known if the final interviewee had a lived refugee experience although they were from a refugee-background community. Table 1 provides an overview of key actor details.

**Table 1:** Summary details of key actors

Interviewee	Community food role	Country of origin/cultural background	From refugee background?
#1 – Community leader	• Paid and unpaid community support (e.g. translation, share cultural store locations) • Provides orientation for new arrivals in the community • Works mainly with Sudanese, but also with many different cultural groups including Thai, Indian, Iranian, Syrian, Afghan, Somali and Iraqi.	Sudan/Ethiopian and Sudanese	Yes
#2 – Community leader	• Sharing of information about where to source cultural foods • Providing cultural dishes to the community • Supports people from all cultural backgrounds in her role	Syria/Armenian Syrian	Yes
#3 – Farmer	• Farming information and advice to other farmers • Provides cultural seeds for other farmers • Provides cultural vegetables to community members • Sells cultural vegetables to broader community members • Advocates for the farm to secure community support • Works mainly with Bhutanese and Nepali communities, but also has contact with Burmese and people from the Solomon Islands at the farm	Bhutan/Bhutanese & Nepali	Yes
#4 – Market stall owner	• Growing food in her backyard and sharing with the community • Selling cultural foods to various cultural communities at the markets, e.g. Thai, Burmese, Lao, Chinese, Pakistani, African communities, “multi”	Cambodia/Cambodian	Yes
#5 – Grocery stall owner	• Sources cultural foods from other countries and imports them into Australia • Sell cultural foods to community members • Works with many African cultures, such as Burundian, Congolese, Rwandese, Tanzanians, Malawian as well as other cultures, such as Filipino, Malaysian and Thai	Australia/Mandingo	Parents from a refugee background
#6 – Restaurant owner	• Selling cultural meals • Education on preparation of meats and dishes from a range of cultures	Afghanistan/Afghan	Unknown, from refugee-background community

#### Data collection

Data were collected on the interviewee’s role and activities supporting cultural food security, including how information was disseminated to the community, perceptions on why the role was important, why the interviewee chose to fill this role and the enablers and barriers they encountered when filling this role. The data collection tool (provided in supplementary materials) was developed by community members during a series of co-design workshops, during which the tool was piloted by holding interviews with four community members, of which one interview is included in the analysis (#1 - community leader).

Interviews were conducted by community researchers, specifically selected based on their ability to speak the language preferred by the key actor. Interviews were held face-to-face at the key actors’ preferred location and in their chosen language. Three interviews were held in English (#1 – community leader; #4 – market stall owner and #5 – grocery stall owner), one in Arabic (#2 – community leader), one in Nepali (#3 – farmer) and one in Dari (#6 – restaurant owner). Interviews lasted between 20 and 45 min, excluding interruptions (33 min on average) except one interview (#6 - restaurant owner) which lasted 5 min. Interviews were audio-recorded and interviewees provided verbal consent at the start of the interview. A gift card (AUD$50) was provided to interviewees to recognise their valuable contribution to the project.

#### Data analysis

Audio recordings of English interviews were translated using Descript^(22)^ and checked for accuracy by the first author. Interviews conducted in other languages (Arabic, Nepali and Dari) were translated and transcribed simultaneously by the community researcher who conducted the interview.

Analysis was undertaken by the first author (TG) and community researchers, of which four are authors (RA, NE, FN and EP). Transcripts were analysed using the ‘best-practice framework for collaborative data analysis^(23)^.’ This framework involves people with lived experiences in the coding framework co-production and includes three phases: preparation; collaborative data analysis (co-production of coding framework) and application (integration, refinement, publication and reflective learning)^(23)^. The preparation phase was completed earlier in the project, where heterogeneous community researchers were hired from preliminary co-design workshops. Prior to analysis, de-identified transcripts were circulated among the community researchers for review. Community researchers met to complete the collaborative data analysis phase, and the following steps were completed for each transcript.

First, the interview setting was described by the community researcher who conducted the interview, and then the group was asked to independently read the transcripts while considering the objectives of the project and their own experiences. Next, a group discussion was held where the community researchers were encouraged to highlight interesting quotes and interpret their meaning, relate this to their own experiences and comment on the significance to communities. They also explored themes across the transcript and, for subsequent transcripts, connections to quotes and discussion points from transcripts already analysed. The first author recorded discussion points on a white board displayed in the room, noting where themes converged. During the application phase, the first author elaborated on discussion points from the previous phase by drawing on quotes from the transcripts and a vignette was created for each interviewee, all of which was then distributed to the research team for further feedback.

## Results

Key actors who were interviewed described filling crucial and unique roles which facilitated access to cultural foods for communities in Greater Brisbane, and their values represented collectivist ideals. A major aspect of these community roles was to empower communities with agency and autonomy over their foodways, including an essential part of aiding settlement for community members. Three themes were identified which supported these findings: fostering improved cultural food security supports settlement; communication methods prioritise experience and improve cultural food security and leadership ethos reflects collectivist values.

### Fostering improved cultural food security supports settlement

Consuming cultural foods generated fond memories of the past, which spanned geographical boundaries to connect people to their home country. Therefore, these actors fostered access to cultural foods which were grown, procured and prepared in a cultural manner. This then facilitated placemaking, a sense of belonging, and ultimately supported the settlement journey.

‘So, if you can play a little role in that, but just bringing the taste here, they can make Australia home as well.’

#5 – Grocery store owner

Food spaces, such as the locations where foods were grown or sold, created a place where community members could connect and nurture their identity and helped create a sense of belonging.

‘Even if I can’t myself do something, I mention that we should do it together as friends, and even then, some don’t, and the garden community supervisors and the … communities people coming together to do something.’

#3 – Farmer

‘Instead of like helping each other, we come and see friends as well. Okay. It’s, it’s more like [a] meeting area. Seeing, like seeing friends that we don’t have time to go and see each other, so they come to the market and then we see them.’

‘Socialising with, you know? Yes. What do you call it? Like it’s the community.’

#4 – Market stall owner

On the other hand, key actors described that a lack of knowledge or ability to influence many Australian food pathways had a negative impact on health and wellbeing. People generally had more trust in foods purchased from cultural stores where they had a better understanding of the production pathways of foods. For example, vegetables that were grown organically, or animals slaughtered using the correct Halal method. In these cultural stores, foods were more likely to be sourced using sustainable and cultural practices, which supported health, identity and placemaking.

‘Cause what we have learned in Australia here, there’s heaps of fertilised food everywhere. And it is not really good for our body and our how we are. We are raised on organic foods, hence why we’re strong, we’re this, and this, and that. But by moving to Australia, we’ve been eating a lot of fed hormone to food and the chickens, all that they’re making us feel … not ourself, basically.’

#5 – Grocery store owner

As a result, increasing cultural food security was an important aspect of these key actors’ roles in supporting the community. Key actors spent significant time and effort sharing cultural foods, and culturally safe and trustworthy sources throughout the community, which then increased cultural food access for the community.

‘One day, in two years, me and my two neighbours. All three of us are Armenians from Aleppo. We went hand-in-hand and took the cars and went to West End, on the bus and used the phone. We didn’t know the place’s name. We knew it was Greek but didn’t know what it was called. We went around and around until we found it. When I found it, I took the card and sent it to all who I know and posted it on Facebook^TM^. Whoever wants the Greek place, this is it.’

#2 – Community leader

### Communication methods prioritise experience and improve cultural food security

Word-of-mouth emerged as the key communication method used by community members when interacting with other community members. This method prioritises the lived experience and knowledge of community members.

‘I can find the information from my friends as well that they have been here long time. And they have a lot of experiences here.’

#1 – Community leader

People in these key community roles used innovative methods to support word-of-mouth communication and the sharing of community knowledge. For example, the community leader discussed how she overcame issues of directing people to new food sources she discovered:

‘I gave them the addresses and cards because I take cards and give it to them, because I don’t know how to direct. So, I take cards and give them away.’

#2 – Community leader

Recent innovations in technology have allowed the digitisation of word-of-mouth communication techniques enabling a broader and more efficient reach to community members. Key actors described using social media platforms in the same manner as they used word-of-mouth communication. For example, posting voice recorded messages in any language to private groups on WhatsApp^TM^ allowed communication to many community members simultaneously, regardless of people’s literacy levels.

‘Yes, in WhatsApp^TM^ group, because, as I told, doesn’t know … how can they write. They send the message as well sometimes. Or how can call you, because doesn’t know your name … but by voice message, they can just click on it and they can listen.’

#1 – Community leader

Word-of-mouth communication improved cultural food security as it was essential for establishing alternative food networks that were not part of the mainstream foodways. People in these community roles networked with other community members to identify trusted business connections, and new cultural food sources and suppliers.

‘Well first someone we knew had known them, and they came and went after which we met, and then they took us and we got to know them. Well, we first got to know the Nepali shop in Carseldine first as that’s where we first sold our greens…. And then a lot of Nepali people started to come and people started to take note of the Nepali greens. Because of that, their shop started to become recognised.’

#3 – Farmer

Communities then shared knowledge among themselves of where cultural foods were available and a new alternative food network was subsequently established.

‘But yeah, just like you just come in and say hello and oh, okay, chat for a while, then we, you know, go … I do get customer and then sometime they bring customer for us.’

#4 – Market stall owner

This cultural food security method also created new food networks across international borders. The grocery store owner described how they established new supply chains of imported cultural foods from other countries by using their customers trusted contacts:

‘When people come in they’ll talk to us, they say what they want and what they like were trying to get, ask and make they know any friend members that they know in that country they can help us [import] that stuff in. And then they might connect us by word of mouth and trusting them. They’ll know that person to do the transaction through.’

#5 – Grocery store owner

### Leadership ethos reflects collectivist values

It was clear that all key actors were important leaders in their community. They were a valuable part of facilitating the settlement journey and were a bridge between and among communities and organisations and across countries and age. The leadership ethos reflected collectivist values, to support the community through inclusivity, helping people and sharing knowledge among community members.

‘Yes, it’s given me a good feeling and it is human work, I think yeah, definitely to helping the people.’

#1 – Community leader

‘It’s because I suffered until I found [cultural foods]. So, they don’t suffer. So, it’s easy. And until now, whenever new Syrians come, I find out who is coming, I tell them whatever [they need].’

#2 – Community leader

‘Any person, straightaway we get to know them, even if you’re not comfortable, you have to be someone that does good. There are a lot of people, for example that I don’t like, but what about them? If there is some help needed, I help and don’t just watch.’

#2 – Community leader

‘I love hearing the feedback [from shoppers] because it makes me feel, it makes my heart feel better cause what I’m doing is right, so it makes me keep doing more… I’m doing something for other people, making them happier, making them smile, putting some joy in their life, which makes me feel better.’

#5 – Grocery store owner

Further, it was important that this ethos was embedded in family values that were passed to children and down through future generations.

‘I’m the kind of person that wherever I am, even my family, my daughters, wherever we are, if we see anyone and someone else with them, it’s not strange for us. Straightaway we say hello and get along with them. So, this is a custom of my family, mine and I taught it to my children.’

#2 – Community leader

Sharing cultural food was highlighted as an important aspect of this ethos, where food was seen as a way of supporting people that extended beyond cultural boundaries.

‘Straightaway I put some away and my eldest daughter came and said “Mum, what is this?” I said “This is for our neighbour, Tina. She has a baby and all that.” Different food. I went straight to her; I yelled out to her and said “This is for you”. I speak a little broken. I said to her: “yoghurt, and chicken, and kibbeh, meat, beef, halal.” She died of laughter.’

#2 – Community leader

Food sharing allowed cultural identity of the community to be celebrated. Key actors interviewed saw their role as a crucial part of community life that facilitated connections and relationships, and food was an important aspect of this connection.

‘This coming and going for many Nepali people happened because of mustard greens or pumpkin greens and others. Maybe around 20 people know and come to me for the greens.’

#3 – Farmer

However, these roles also came with a burden of community responsibility. Key actors were often engaging in unpaid work in the community in addition to their paid employment.

‘And I do it as well now because sometimes we are working as five hour or six hours. And as the time we are working as a volunteer. Even sometimes until evening times. … Community work is a lot of [volunteering].’

#1 – Community leader

This may be exacerbated by service providers that may not understand the importance of cultural food security in settlement. Therefore, these service providers may not have shown new arrivals where to find culturally appropriate and trustworthy grocery stores and instead relied on community members to provide this essential information.

‘I wanted our food. How are we supposed to do this? So, the people at TAFE [Technical and Further Education], the people at the welcome house were also in TAFE. In the afternoon, they came back from TAFE. We got to know them, and we were in welcome house for 2 months. Two months. We got to know them and taught us the Arab shops from Kuraby to [fruit store], to all of it. Where the shops are, where the vegetables are, where everything is, we learned it all.’

#2 – Community leader

There may have also been additional pressures on other community members to ensure culture was maintained. The farmer discussed how food sharing practices in her community was different in Australia. The responsibilities to prepare and share cultural foods as part of cultural celebrations were amplified, especially for young children who had not experienced culture in their home country.

‘Well instead of doing things within your own family and home [like we used to in Nepal], within the community most events are done together at a venue, like a hall with the other Bhutanese people. To show and share our culture they do and are doing it still. At home [in Nepal], it doesn’t matter what we do or to what extent we do it, but within the community [in Australia] we have to make many types of cultural foods.’

#3 – Farmer

## Discussion

Similar to other studies^(1–3,7)^, this research found that fostering improved cultural food security created connections across geographical locations and cultures, functioning as devices for placemaking generating a sense of belonging and supporting the settlement journey. Foodways that used cultural methods for sourcing and preparation of foods were generally trusted over more mainstream foodways. This study also found key community roles were integral to facilitating cultural food security and places where cultural foods were available (grown or sold) were considered community assets that facilitated community and connection.

Communities utilised communication methods that prioritised the knowledge, wisdom and experience of community members. Community members sought and shared the opinions of other trusted community members and used word-of-mouth (or a digitised version) to spread knowledge of new foodways throughout the community. This communication method was used as a business tool to source and secure customers and supplies across international boundaries. These practices resulted in alternative food networks that sustained cultural food security for communities in Greater Brisbane.

These new alternative food networks indicate a collective agency over foodways, which suggests increased food sovereignty. Food sovereignty is ‘the right of peoples to healthy and culturally appropriate food produced through ecologically sound and sustainable methods, and their right to define their own food and agriculture systems^(24)^.’ The inclusion of ‘culturally appropriate food’ in the definition suggests food sovereignty places value on ensuring people have agency over foodways that support their culture and identity. Cachelin and colleagues^(15)^ states ‘(food sovereignty) implies we should see food as a set of relationships rather than as a commodity’. This shifts the focus away from the nutritional content of food to a spiritual and cultural emphasis, which was seen in the results of this study. Food sovereignty also means food systems that support the longevity and health of communities by nurturing and caring for community resources, such as land and seas^(8)^. This study further found that all food spaces (e.g. where foods are sold) are community resources that should be cared for.

Current food production pathways in Australia are often complex and involve many intermediaries from fisheries and agriculture, warehousing and storage, transport and distribution, processing and retail industries. Globalisation has meant these pathways are increasingly under corporate control, and hence economically driven^(25)^. This commercialisation is underpinned by individualistic ideals that value self-sufficiency and autonomy, where land and food are seen as commodities^(15,16)^. In Australia, these corporations regularly lobby policy-makers to influence food policies that support financial objectives^(26)^. Food sovereignty, however, is underpinned by social justice and human rights and advocates for democratised food systems^(15,27)^. It requires a radical shift to transfer the decision-making and influence of financially driven corporations to farmers, First Nations peoples, food workers and community members^(25)^.

This study found community members were able to influence the decisions of smaller retailers (that had a collectivist agenda) to increase the supply of cultural foods, indicating a level of food sovereignty. Other researchers have found similar outcomes. For example, people from refugee backgrounds reported accessing fresh and healthy foods from cultural grocery stores even though they were living in a location identified as a ‘food desert^(15)^.’ People generally trust stores run by community members (which includes the broader diaspora of those with a lived refugee or migrant experience) to provide their foods^(3,28)^, and these stores may be more willing to accommodate requests for new products^(15)^, thereby creating new foodways. Over time, people may help shape their food environment by introducing new foods and sharing those foods with community members^(1,3)^. This implies that people with lived refugee experiences actively exert food sovereignty by establishing alternative food networks that bypass other food production pathways.

Finally, this study found community members in leadership roles had an ethos that reflected collectivist values. The needs of the community were important as part of their own health and well-being, and these beliefs were passed down through generations. Similar to other studies^(1,3,29–31)^, it was found that sharing cultural food was seen as a way of supporting the community that extended beyond cultural boundaries and facilitated connections and relationships. However, leadership roles also came with a level of responsibility. It may have compelled leaders to carry out unpaid volunteer work and ensure culture was honoured and respected through public celebrations of cultural and religious events.

The main strength of this study was the community-led methodology, participatory action research. Community members were involved in co-designing the research methods and tools and conducting the interviews. This ensured the study was culturally appropriate and trauma informed. Community members were also involved in data analysis, which may have reduced the likelihood of biases impacting on data interpretation. The main limitation of this study design was the small sample size of six interviewees and therefore may not represent the views and values from the diverse range of communities found in Greater Brisbane.

### Conclusion

This study sought to explore key community roles in refugee-background communities to understand how they supported cultural food security. This provided a unique perspective on how communities were inherently structured to support each other. It showed that people from refugee backgrounds take collective action over their foodways, and leadership roles retain collectivist values that are instrumental to supporting cultural food security. This collective action has the hallmarks of food sovereignty where food justice and access are prioritised over profits. Researchers and public health workers should work together with communities and recognise community expertise and agency over foodways to create interventions targeting cultural food security and autonomy. Furthermore, policy makers should engage with key actors within communities to ensure policy is relevant and acts to build food sovereignty to foster community health and well-being.
